# The phenomenology of psilocybin’s experience mediates subsequent persistent psychological effects independently of sex, previous experience, or setting

**DOI:** 10.1007/s43440-025-00742-5

**Published:** 2025-06-16

**Authors:** Tereza Klučková, Marek Nikolič, Filip Tylš, Vojtěch Viktorin, Čestmír Vejmola, Michaela Viktorinová, Anna Bravermanová, Renáta Androvičová, Veronika Andrashko, Jakub Korčák, Peter Zach, Kateřina Hájková, Martin Kuchař, Marie Balíková, Martin Brunovský, Jiří Horáček, Tomáš Páleníček

**Affiliations:** 1 National Institute of Mental Health, Topolová 748, Klecany, 250 67 Czechia; 2 Third Faculty of Medicine, Charles University Prague, Ruská 87, Prague, 100 00 Czechia; 3 Forensic Laboratory of Biologically Active Compounds, Department of Chemistry of Natural Compounds, University of Chemistry and Technology, Prague, Czechia; 4 First Faculty of Medicine, Charles University Prague, Kateřinská 32, Prague, 121 08 Czechia

**Keywords:** Psilocybin, Repeated administration, Healthy population, Previous experience, Sex

## Abstract

**Background:**

Recent studies intensively explore psilocybin’s antidepressant potential, but variables like previous experience, repeated use, setting, and sex remain underexplored. This study examines acute and long-term effects of psilocybin in healthy individuals.

**Methods:**

A double-blind, placebo-controlled, cross-over study included 40 healthy participants (20 females, mean age 38). Each received two doses of psilocybin (0.26 mg/kg) at least 56 days apart (mean 488) in two neuroimaging study arms. Nearly half had previous psychedelic experience. Acute effects were measured using the Altered States of Consciousness Scales (ASCs) and a Visual Analogue Scale (VAS) for emotional valence. The Persisting Effects Questionnaire (PEQ) assessed long-term effects.

**Results:**

All results were independent of observed variables such as previous psychedelic experience, repeated use, setting, sex and occupation. Acute effects were moderate on the ASCs, with VAS ratings showing mostly pleasant or fluctuating experiences and only one unpleasant session. All experiences resolved in a positive or neutral state by the session’s end. Psilocybin produced lasting positive effects across all PEQ domains, with negligible negative effects. Oceanic Boundlessness (OBN) and Visionary Restructuralization (VRS) correlated with positive outcomes, while Dread of Ego Dissolution (DED), typically associated with fear, did not predict negative effects. The nature of the acute experience (pleasant or mixed) was not linked to the direction or intensity of long-term outcomes. Peak experiences ending in a positive mood were strongly associated with favourable long-term effects.

**Conclusion:**

Repeated psilocybin administration in healthy individuals induces positive, lasting effects, with challenging experiences in controlled settings not causing adverse outcomes. These findings support psilocybin’s psychological safety and its repeated use in clinical trials.

**Supplementary Information:**

The online version contains supplementary material available at 10.1007/s43440-025-00742-5.

## Introduction

Psilocybin, a natural serotonergic psychedelic of the tryptamine family, has recently gained robust evidence as a potential antidepressant [1–4]. Other studies also suggest its benefits in terms of positive, sustained changes in personal well-being and attitudes towards self and others in healthy subjects [5–7]. Unlike classical antidepressants, psilocybin’s antidepressant effects occur almost immediately after a sufficiently potent dose. The onset of action is comparable to that of the dissociative anaesthetic, NMDA (N-methyl-D-aspartic acid) antagonist, ketamine, which is now widely used off-label as an antidepressant in patients [8, 9]. Because of these rapid effects, psilocybin, ketamine, and other psychedelic drugs have been combined into a new class of so-called rapid-onset antidepressants [10–12].

One of the key questions regarding the neurobiology of the antidepressant effects of psilocybin and other psychedelics is the extent to which the psychedelic experience drives the antidepressant effect. While there are some indicators of the importance of psychedelic or psychotomimetic effects, or the peak and mystical type of experience, for the long-term positive outcomes of psilocybin and ketamine in both healthy and clinical populations [3, 13–23], other research, with ketamine and esketamine, suggests that similar effects can be achieved only pharmacologically, bypassing the psychedelic state [24–29]. Subsequent critical questions concern the consistency of these positive long-term effects with repeated administrations and their influence on phenomenology. In addition, the influence of other variables, such as the role of sex, set, and setting, warrants investigation.

The psilocybin-induced experience is characterised by perceptual, emotional, and cognitive alterations and a subjective loss of identity, described as ego dissolution. The positive nature of ego dissolution in terms of being a predictor of beneficial psychological effects is often referred to as a peak [30], mystical [31–33], or enlightening experience [34]. It is thought to be one of the most important factors in determining long-term outcomes following psychedelic treatment [35, 36]. These acute effects of psilocybin are typically assessed using Altered State of Consciousness scales (ASCs), the Mystical Experience Questionnaire (MEQ) or Visual Analogue Scale (VAS) [37]. The intensity and positive valence of the psychedelic experience when culminating in a ‘peak’ experience, seems to predict beneficial long-term outcomes [21, 38], while intensive anxiety seems to be associated with the opposite [21]. The proposed mediators of the effect of peak experiences on long-term outcomes are the increase in psychological flexibility [39, 40], awe [41], and changes in brain criticality [42, 43]. Previous findings in healthy subjects have shown that repeated psychedelic experiences result in less acute effects [44, 45] and found no effect of sex. However, in contrast to acute effects, there is no evidence on the role of these factors on long-term outcomes.

Given these considerations, the primary objective of this study is to elucidate the relationship between acute phenomenological experiences and potential antidepressant-like effects in healthy subjects. Specifically, the study aims to examine the correlation between phenomenology and the enduring effects of the psilocybin experience, accounting for variables such as previous experience with psychedelics, repeated administration, and sex. Unlike in patient populations, assessing antidepressant-like effects in healthy individuals requires an emphasis on more nuanced alterations, including overall well-being and mood shifts, which may not attain a level of pathological significance. Such persistent changes are captured by the Persisting Effects Questionnaire (PEQ) [5, 6], which effectively measures attitudes about self and life, mood, relationships, behaviour, and spirituality. The PEQ was constructed based on previous research that reported positively valued psilocybin experiences in healthy volunteers [46], which had been shown to have personal significance even after 25 years [47]. The PEQ also showed optimal convergent validity with the phenomenology of the experience as assessed by the ASCs and its renewed version, Five Dimensional Altered States of Consciousness (5D-ASC) [48–50].

Thus, here we tested the effects of the acute phenomenology of the psilocybin experience, assessed by ASCs and VAS describing the course and directionality of the emotional valence of the experience, on the long-term effects on mood, well-being and spirituality, assessed by PEQ, in healthy subjects, controlled for previous experience with psychedelics, treatment order and sex. As the data presented here come from a larger study that is also evaluating the neurobiology of psilocybin’s effects using electroencephalography (EEG) and functional magnetic resonance imaging (fMRI) perspective, we have collected data from participants exposed to psilocybin repeatedly in two different settings (EEG and fMRI).

Based on the literature, we hypothesize that the psilocybin-induced psychedelic experience will drive long-term positive outcomes, while the above variables will be treated as exploratory measures.

## Materials and methods

### Study approval

The study was conducted at the National Institute of Mental Health (formerly the Prague Psychiatric Center). It was approved on 18.6.2014 under reference number 60/14 by the Internal Review Board (IRB) entitled “Ethical Committee of the Prague Psychiatric Centre”, which subsequently became the “Ethical Committee of the National Institute of Mental Health”, which was in charge for the remainder of the study. The study was conducted in accordance with the Declaration of Helsinki. Each participant was fully informed and gave full consent to the study procedures by signing the “Information for Volunteers and Informed Consent to Participate in a Clinical Research Study”. It was approved by the State Institute for Drug Control on December 11, 2013, under registration number 92/13. The study was registered as a clinical trial under EudraCT No. 2012-004579-37.

### Clinical trial registration

Clinical trial registration: EudraCT 2012-004579-37, https://www.clinicaltrialsregister.eu/ctr-search/trial/2012-004579-37/CZ.

### Experimental design

**Fig. 1:**
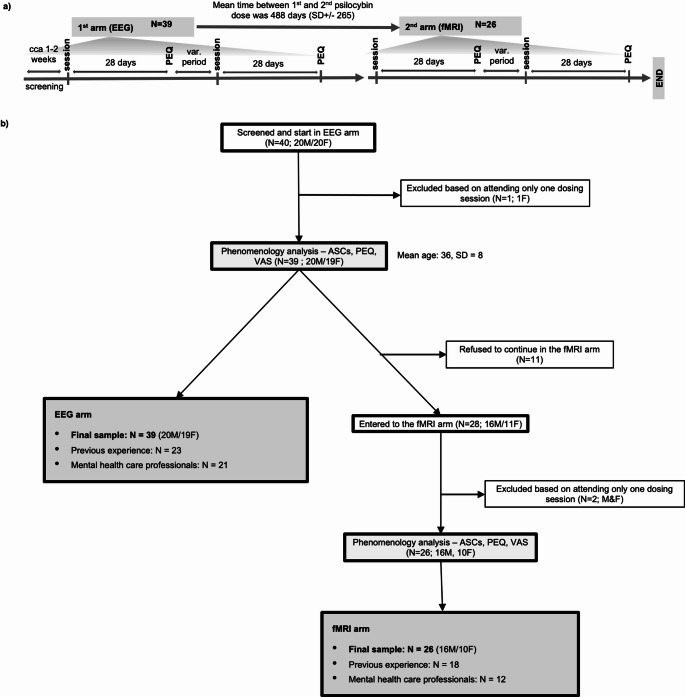
**(a)** Experimental timeline of the two-arm design. After the screening participants first underwent the EEG arm (*N* = 39), followed by the fMRI arm (*N* = 26). In each arm, participants received both treatments—psilocybin and placebo—in a double-blind, crossover design. The order of administration was balanced across participants. The minimum interval between sessions was 28 days. The mean interval between the first and second psilocybin session was 488 days (SD ± 265). **(b)** Study flowchart shows screening, inclusion, and final sample characteristics of the two study arms including biological sex, prior psychedelic experience, and occupation. Only participants who completed both psilocybin and placebo sessions and all key psychometric assessments (ASCs, PEQ, VAS) were included in the final analysis. 40 participants were included in the study. One participant was excluded from the EEG arm for completing only one session (final *N* = 39). In the fMRI arm, 11 participants withdrew, and 2 completed only one session (final *N* = 26). Abbreviations: ASCs - Altered States of Consciousness Scales, EEG - Electroencephalography, F - Female, fMRI - Functional Magnetic Resonance Imaging, M - Male, PEQ - Persisting Effects Questionnaire, VAS - Visual Analogue Scale. Data were collected at the National Institute of Mental Health (formerly the Prague Psychiatric Center) between 2013 and 2020. Clinical trial registered under EudraCT No. 2012-004579-37

The primary objective of the study was neuroimaging investigation. Data were collected in two consecutive arms in which subjects underwent a battery of questionnaire-based, phenomenological, neurocognitive, and EEG or fMRI measurements. While the first arm was designed to collect primarily EEG data (EEG arm), the second arm was designed to collect primarily fMRI or simultaneous EEG/fMRI measurements (fMRI arm). As the study allowed for the enrolment of inexperienced subjects, the EEG arm always preceded the fMRI arm, as we agreed that the procedures associated with EEG were less stressful, so that we did not expose inexperienced subjects directly to the demanding environment of fMRI. In each arm, subjects received both treatments, psilocybin and placebo, in a double-blind crossover design. Each participant thus completed four dosing sessions in total — one with psilocybin and one with a placebo in each arm. The order of administration was balanced across participants to control for order effects. The minimum interval between sessions was 28 days. See Fig. 1a for details of the experimental timeline.

Throughout the study, each participant was accompanied by a fixed pair of sitters consisting of one man and one woman, one of whom was a psychiatrist. Prior to dosing, details of the study procedures and drug effects were discussed during a preparatory session. Each dosing session began with a brief clinical examination to check inclusion/exclusion criteria. This included a somatic examination, questions about a current living situation, blood pressure and heart rate, and a negative result on a breathalyser test and a urine drug screen. An intravenous cannula was inserted for blood sampling and an EEG cap was placed on the participant’s head before the session. The 6–8 h session, starting at 7–8 am, took place in a decorated EEG experimental room, where the subjects spent most of the experiment. In the case of the fMRI arm, subjects went to an MRI scanner in a neighbouring laboratory on three occasions (baseline, 90 and 240 min after dosing) for a 45–60 min scanning session. The treatments used were oral psilocybin 0.26 mg/kg and placebo (Tritici Amylum). The formulations - capsules containing either 5 or 1 mg of psilocybin - were prepared in a pharmacy at IKEM (Institute of Clinical and Experimental Medicine in Prague). The dose of psilocybin was adjusted by combining capsules containing 1 and 5 mg, increasing or decreasing by 1 mg per 5 kg of body weight, with a 75 kg person being treated with 20 mg of psilocybin. In the case of placebo, the number of capsules was identical. The average dose used in the study was 18.6 mg (15–24 mg). Subjects took the capsules on an empty stomach with 200 ml of water. The study nurse was also present throughout the session in a neighbouring room. The women’s sessions were scheduled outside the menstrual phase of the female cycle to reduce interindividual variation due to the effect of menstruation and to avoid potential differences associated with different phases of the cycle [51]. After the acute effects had subsided, participants completed questionnaires assessing the quality of the experience. At the follow-up, 28 days after dosing, participants were invited to complete a questionnaire on the persistent effects attributed to their last session.

### Participants

Recruitment was carried out using a peer-to-peer snowball sampling method. It started with an initial pre-screening by telephone interview for key exclusion criteria (Table S1 in the online supplement). The study was designed to balance the whole group in terms of sex (female/male) and previous experience with psychedelics. Although this was not the primary aim, the snowballing method resulted in a near-balanced sample in terms of mental health and non-mental health education/work status. Eligible participants were invited to a face-to-face interview. Participants were informed of the study design, the effects of psilocybin, safety concerns, and that they were free to withdraw from the study at any time. Each participant was given space to ask questions and gave full consent to the study procedures by signing the “Information for Volunteers and Informed Consent to Participate in a Clinical Research Study”. Participants were screened using the Minnesota Multiphasic Personality Inventory 2 (MMPI-2) [52] and the Mini-International Neuropsychiatric Interview version 5.0 (MINI v 5.0) [53] to rule out psychopathology and were physically examined to rule out somatic problems [54]. 40 participants were included in the study. See Fig. 1 for the exclusion process and the final sample. 26 subjects participated in both the EEG and fMRI arms, as 14 subjects did not continue in the fMRI part of the study. The main reason for not continuing was that they were afraid of the fMRI setting and tasks, which they found to be disturbing and a long time frame between the EEG and fMRI arm. Details of the enrolment process and sample characteristics are shown in the flowchart in Fig. 1b and in Table 1.

**Table 1:** Sample characteristics (*N* = 39). Data are mean ± sd, or N. One participant did not report occupation and previous experience

Variable	Males *N* = 20	Females *N* = 19
Age	35.55 ± 7.44	36.05 ± 7.84
Occupation		
MH	9	12
O	11	7
Experience with substances		
E	14	9
Na	6	10

Abbreviations: MH - Mental health occupation; O – Other occupation; E – Experienced (previous experience with psychedelics); Na – Naïve (without previous experience with psychedelics)

Data were collected at the National Institute of Mental Health (formerly the Prague Psychiatric Center) between 2013 and 2020. Clinical trial registered under EudraCT No. 2012-004579-37

### Altered states of consciousness scales (ASCs)

Immediately after the session, the participants completed the Czech version of the ASCs questionnaire (in the original: Aussergewöhnliche Psychische Zustände APZ) [48]. It consists of 72 questions assessing the subjective acute effects of the altered state of consciousness in 3 subscales and 1 total scale including all questions: Oceanic Boundlessness (OBN), Dread of Ego Dissolution (DED) and Visionary Restructuralization (VRS), and the General Altered States of Consciousness (GASC).

The OBN scale refers to a deeply positive experience of depersonalisation and oneness. High scores indicate a mystical experience of oneness. The DED scale refers to a negative experience of depersonalisation, loss of control and increased anxiety. High scores indicate a difficult and probably unpleasant nature of the experience, which, if it persists throughout the experience, can be referred to as a bad trip in an uncontrolled environment. The VRS scale refers to perceptual alterations and includes symptoms such as illusions, (pseudo) hallucinations, synesthesia, typically but not limited to the visual domain. The secondary scale GASC consists of all 72 items of the questionnaire and can be interpreted as a general measure of altered consciousness.

The original ASCs scale was used as it was, at the time of study design, the most widely employed tool for assessing altered states of consciousness. This choice enabled comparison with existing studies and ensured continuity in the local research context.

### Persisting effects questionnaire (PEQ)

The PEQ [5, 6] consists of 140 questions in 6 categories, which are subsequently divided into positive and negative categories: attitudes about self, attitudes about life, mood changes, altruistic/social effects, behavioural changes, and spirituality; and 3 additional questions on personal meaning, spiritual meaning and change in life satisfaction. Most items are rated on a 6-point scale (0 = not at all; 1 = so slight cannot decide; 2 = slight; 3 = moderate; 4 = strong; 5 = extreme, more than ever before in your life and stronger than 4). The three additional questions are: (1) How personally meaningful was the experience? (Rated from 1 to 8, with 1 = no more than routine, everyday experiences; 7 = among the five most meaningful experiences of my life; and 8 = the single most meaningful experience of my life); (2) Indicate the degree to which the experience was spiritually significant to you? (Rated from 1 to 6, with 1 = not at all; 5 = among the five most spiritually significant experiences of my life; 6 = the single most spiritually significant experience of my life); (3) Do you believe that the experience and your contemplation of that experience have led to change in your current sense of personal well-being or life satisfaction? (Rated from + 3 to − 3, with + 3 = increased very much to – 3 = decreased very much)

### Visual analogue scale (VAS)

At the end of the dosing session, subjects were asked to plot the course of the pleasant/unpleasant emotional valence of their experience on the VAS as a continuous curve over 7 hours of experience on a 2D graph (time from 0 to 7 in hours was on the X-axis; Y-axis = pleasant/unpleasant experience, VAS range 10 to -10), according to the following task “Plot a continuous graph of how your intoxication progressed in terms of pleasant and unpleasant (anxious) experiences [55]. Use the highest values only for ecstatic states or states of opposite extreme polarity. The plotted forms were then digitised using the freely available tool Webplotdigitizer with the algorithm “X step w/ Interpolation” with a step size of 0.1 and a smoothing of ΔX of 200%.

### Exploratory analyses

We examined the associations between the VAS and PEQ. Based on a closer look at our data, we have tested whether subjects with ‘pleasant only’ and ‘oscillating between pleasant and unpleasant’ experiences would differ in their persisting effects. Subsequently, we also evaluated whether the peak of the experience or the end point of the experience or their combination would be associated with the long-term outcome [21].

### Psilocin serum levels

Blood samples were taken before the experiment and at 1, 2, 3 and 6 h after dosing. During the study, due to external conditions, we had to change the laboratory performing the measurements of psilocin serum levels, switching from earlier measurements performed by the gas chromatography–mass spectrometry (GC-MS) (first 20 subjects in the EEG arm) [54] to the remaining measurements performed by liquid chromatography–mass spectrometry (LC-MS) [56].

### Data processing and statistical analyses

Descriptive statistics were calculated. Independent variables were sex, previous experience, and order^1^. Dependent variables were ASCs and PEQ scores. Normality of the data was assessed visually (QQ plots) and statistically (Shapiro-Wilk test). PEQ and ASCs scores were normalised to percentages to facilitate visual understanding. Samples were also matched, and paired data was analysed. Data were tested for equal variances (Levene test). The difference between psilocybin and placebo, and the first and second psilocybin session was compared (Paired-sample Student’s t-test, equal variances not assumed). Benjamini-Hochberg correction (BH) was applied.

Separate ANOVA Repeated-Measures model was fit for each dependent variable (DV; DED, OBN, VRS, and GASC, PEQ Positive Subscales) using the aov() function in R. Model controlled for the effect of previous experience, work (mental health specialist or not), order of experience (psilocybin or placebo first with the session), and sex. Repeated design was considered using the error term participants $\:ID\:*\:\left(Session\:*\:Substance\right)$, where ID was individual identification and session was EEG or MRI. The model formula was:

$$\begin{aligned}\:DV\:\sim\:&Substance+Experience+Work+Order+Sex\\&+\left(ID*\left(Session*Substance\right)\right)\:\end{aligned}$$

Pairwise comparisons were conducted as a post hoc test. Correction between models was not performed.

The Pearson correlation between ASCs and PEQ subscales was calculated and corrected using the BH procedure.

VAS data were integrated into the total area under the curve (AUC) and the total positive area under the curve (only values above zero; pAUC). The relative index of positive experience (RIPE) was calculated as the ratio of AUC to pAUC. The RIPE index was used to divide the sample into those who had only positive experiences and those who had both positive and negative experiences. This new factor was then used in a secondary analysis of ASCs and PEQ. A two-sample Student’s t-test was used *p-values* were not corrected.

In an exploratory analysis, we explored the difference between participants who experienced only positive, and both positive and negative emotions during the experience with an independent sample Student’s t-test, equal variances not assumed. *P-values* uncorrected. Finally, the average of the highest VAS score and a score at 6 h was calculated for each participant (peak-end rule) [57] and correlated with the dependent variables, *p-values* uncorrected.

Statistical analysis was performed using RStudio software for iOS, version 1.4.1743 (RStudio, Inc., 2009–2022).

## Results

### Sample characteristics and psilocin serum levels

The sample characteristics, including participant demographics such as age, sex distribution, and previous experience with psychedelics, are comprehensively detailed in Fig. 1; Table 1. The table also provides statistical information on the mean age. Psilocin serum levels, measured at multiple time points after psilocybin administration, are illustrated in Fig. S5 in the online supplement.

### Altered states of consciousness scales (ASCs)

**Fig. 2:**
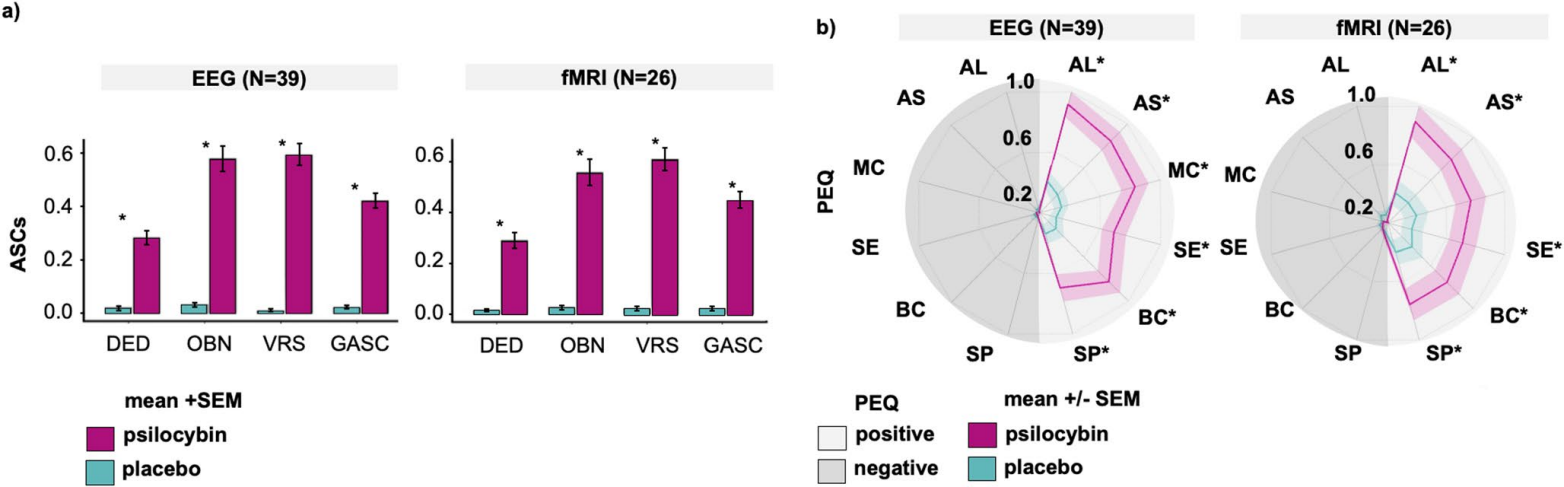
**(a)** Psilocybin induced a significant increase in all domains of the Altered States of Consciousness (ASCs) questionnaire. **(b)** Psilocybin induced a significant increase in all positive subscales of the Persisting Effects Questionnaire (PEQ). The effect on negative subscales was negligible. The difference between psilocybin and placebo for each each subscale was compared using a paired-sample Student’s t-test, *p-values* adjusted using the Benjamini-Hochberg correction. * indicates *p* < 0.05. In **b)**, positive subscales are displayed in the light grey, and negative subscales in the dark grey. **Abbreviations**: AL - Attitudes towards life, AS - Attitudes toward self, ASCs - Altered States of Consciousness Scales, BC - Behavioral changes, DED - Dread of Ego Dissolution, EEG - Electroencephalography, fMRI - Functional Magnetic Resonance Imaging, GASC - General Altered States of Consciousness, MC - Mood changes, OBN - Oceanic Boundlessness, PEQ - Persisting Effects Questionnaire, SEM - Standard Error of the Mean, SE - Social effects, SP - Spirituality, VAS - Visual Analogue Scale, VRS - Visionary Restructuralization. Data were collected at the National Institute of Mental Health (formerly the Prague Psychiatric Center) between 2013 and 2020. Clinical trial registered under EudraCT No. 2012-004579-37

There was a significant difference between the psilocybin and placebo conditions in both the EEG and fMRI arms (Fig. 2a, c, means and standard deviations (SD) are presented in Table S2 in the online supplement). Student’s t-test showed a difference on each subscale (Table 2, *p* < 0.001, *p-values* adjusted for multiple comparisons). The ANOVA model showed a significant effect of treatment on each subscale. We did not find a significant effect of sex, previous experience, occupation, or difference between the EEG-fMRI sessions, represented by the interaction term Treatment: Session (Table 3).

**Table 2:** Results of paired-sample student’s t-tests comparing psilocybin and placebo conditions on subscales of the altered States of consciousness scales (ASCs). The table reports *t-values* with degrees of freedom (df) and *p-values* adjusted for multiple comparisons using the Benjamini–Hochberg correction

Variable	DED	OBN	VRS	GASC
EEG	*t*_*38*_ *=* 36.58	*t*_*38*_ *=* 55.47	*t*_*38*_ *=* 66.24	*t*_*38*_ *=* 50.28
	*p* < 0.001	*p* < 0.001	*p* < 0.001	*p* < 0.001
fMRI	*t*_*25*_ *=* 38.62	*t*_*25*_ *=* 59.02	*t*_*25*_ *=* 71.89	*t*_*25*_ *=* 59.19
	*p* < 0.001	*p* < 0.001	*p* < 0.001	*p* < 0.001

Abbreviations: DED - Dread of Ego Dissolution, EEG – Electroencephalography, fMRI – Functional magnetic resonance imaging, GASC - General Altered States of Consciousness, OBN - Oceanic Boundlessness, VRS - Visionary Restructuralization

Data were collected at the National Institute of Mental Health (formerly the Prague Psychiatric Center) between 2013 and 2020. Clinical trial registered under EudraCT No. 2012-004579-37

**Table 3:** Results of repeated measures ANOVA on altered States of consciousness (ASCs) subscales. A separate model was fitted for each dependent variable (ASCs subscales). ^1^psilocybin vs. Placebo. ^2^order of administration of psilocybin and placebo

Independent variable	DED	OBN	VRS	GASC
Treatment^1^	F_1,35_ = 114.31	F_1,35_ = 150.95	F_1,35_ = 321.43	F_1,35_ = 234.42
	*p* < 0.001	*p* < 0.001	*p* < 0.001	*p* < 0.001
Occupation	F_1,35_ = 0.36	F_1,35_ = 0.12	F_1,35_ = 1.65	F_1,35_ = 0.03
	*p* = 0.55	*p* = 0.72	*p* = 0.27	*p* = 0.86
Previous Experience	F_1,35_ = 1.95	F_1,35_ = 1.20	F_1,35_ = 0.44	F_1,35_ = 1.34
	*p* = 0.17	*p* = 0.28	*p* = 0.51	*p* = 0.25
Order^2^	F_3,35_ = 0.22	F_3,35_ = 0.35	F_3,35_ = 0.90	F_3,35_ = 0.23
	*p* = 0.87	*p* = 0.79	*p* = 0.45	*p* = 0.87
Sex	F_1,35_ = 0.04	F_1,35_ = 0.08	F_1,35_ = 0.14	F_1,35_ = 0.22
	*p* = 0.84	*p* = 0.78	*p* = 0.71	*p* = 0.64
Interaction Treatment x Session	F_24,35_ = 0.17	F_24,35_ = 0.19	F_24,35_ = 0.33	F_24,35_ = 0.18
	*p* = 0.99	*p* = 0.99	*p* = 0.99	*p* = 0.99

Abbreviations: ASCs - Altered States of Consciousness, DED – Dread of Ego Dissolution, GASC – General Altered States of Consciousness, OBN – Oceanic Boundlessness, VRS – Visionary Restructuralization

Data were collected at the National Institute of Mental Health (formerly the Prague Psychiatric Center) between 2013 and 2020. Clinical trial registered under EudraCT No. 2012-004579-37

### Persisting effects scale (PEQ)

We found a significant difference between the psilocybin and placebo conditions for all positive subscales (Fig. 2b; Table 4; *p* < 0.001, *p-values* adjusted, means and SD in the online supplement Table S3). ANOVA showed a significant effect of treatment between psilocybin and placebo. We did not find the effect of occupation, previous experience, sex, administration order, or difference between first and second sessions (Table 5, repeated design represented by the interaction term Treatment: Session).

13 out of 39 participants (33%) rated the psilocybin experience as one of the top 5 most meaningful experiences of their lives. The experience increased the current sense of personal well-being or life satisfaction moderately for 14 participants (36%) and very much for 10 participants (26%). 6 participants (15%) rated the psilocybin experience as the single most spiritually significant experience of their lives, and a further 8 participants (21%) rated it among the top five most spiritually significant experiences.

**Table 4:** Results of paired-sample student’s t-tests comparing psilocybin and placebo conditions across subscales of the persisting effects questionnaire (PEQ) in the EEG and fMRI arms. The table reports *t-values* with degrees of freedom (df) and *p-values* adjusted using the Benjamini–Hochberg correction

	Attitudes about life	Attitudes about self	Mood changes	Behavior changes	Altruistic/positive social effects	Increased spirituality
EEG	*t*_38_ = 29.11	*t*_38_ = 25.29	*t*_38_ = 25.97	*t*_38_ = 21.22	*t*_38_ = 24	*t*_38_ = 18.46
	*p* < 0.001	*p* < 0.001	*p* < 0.001	*p* < 0.001	*p* < 0.001	*p* < 0.001
fMRI	*t*_25_ = 16.62	*t*_25_ = 14.39	*t*_25_ = 12.92	*t*_25_ = 12.19	*t*_25_ = 10.8	*t*_25_ = 12.28
	< 0.001	< 0.001	< 0.001	< 0.001	< 0.001	< 0.001

Abbreviations: EEG - Electroencephalography, fMRI - Functional magnetic resonance imaging

Data were collected at the National Institute of Mental Health (formerly the Prague Psychiatric Center) between 2013 and 2020. Clinical trial registered under EudraCT No. 2012-004579-37

**Table 5:** Results of repeated measures ANOVA on persisting effects questionnaire (PEQ) subscales. A separate model was fitted for each dependent variable (PEQ subscales). ^1^psilocybin vs. Placebo. ^2^order of administration of psilocybin and placebo

	Attitudes about life	Attitudes about self	Mood changes	Behavior changes	Altruistic/positive social effects	Increased spirituality	
Treatment^1^	F_1,35_=29.53	F_1,35_=23.60	F_1,35_=22.46	F_1,35_=18.11	F_1,35_=15.90		F_1,35_=10.51
	*p* < 0.001	*p* < 0.001	*p* < 0.001	*p* < 0.001	*p* < 0.001		*p* = 0.002
Occupation	F_1,35_=0.08	F_1,35_=0.02	F_1,35_=0.02	F_1,35_=0.73	F_1,35_=0		F_1,35_=0.16
	*p* = 0.77	*p* = 0.90	*p* = 0.96	*p* = 0.40	*p* = 0.96		*p* = 0.69
Previous Experience	F_1,35_=0.34	F_1,35_=0.96	F_1,35_=0.96	F_1,35_=0.02	F_1,35_=0.68		F_1,35_=0.35
	*p* = 0.56	*p* = 0.33	*p* = 0.33	*p* = 0.88	*p* = 0.42		*p* = 0.55
Order^2^	F_3,35_=0.33	F_3,35_=0.36	F_3,35_=0.15	F_3,35_=0.12	F_3,35_=0.16		F_3,35_=0.03
	*p* = 0.80	*p* = 0.77	*p* = 0.93	*p* = 0.91	*p* = 0.92		*p* = 0.99
Sex	F_1,35_=0.56	F_1,35_=1.91	F_1,35_=0.65	F_1,35_=0.02	F_1,35_=1.33		F_1,35_=1.56
	*p* = 0.45	*p* = 0.17	*p* = 0.42	*p* = 0.88	*p* = 0.26		*p* = 0.93
Treatment: Session	F_24,35_=0.23	F_24,35_=0.27	F_24,35_=0.31	F_24,35_=0.49	F_24,35_=0.13		F_24,35_=0.09
	*p* = 0.99	*p* = 0.99	*p* = 0.99	*p* = 0.96	*p* = 0.99		*p* = 1

Abbreviations: PEQ - Persisting Effects Questionnaire

Data were collected at the National Institute of Mental Health (formerly the Prague Psychiatric Center) between 2013 and 2020. Clinical trial registered under EudraCT No. 2012-004579-37

### Correlation between ASCs and PEQ: positive

**Fig. 3:**
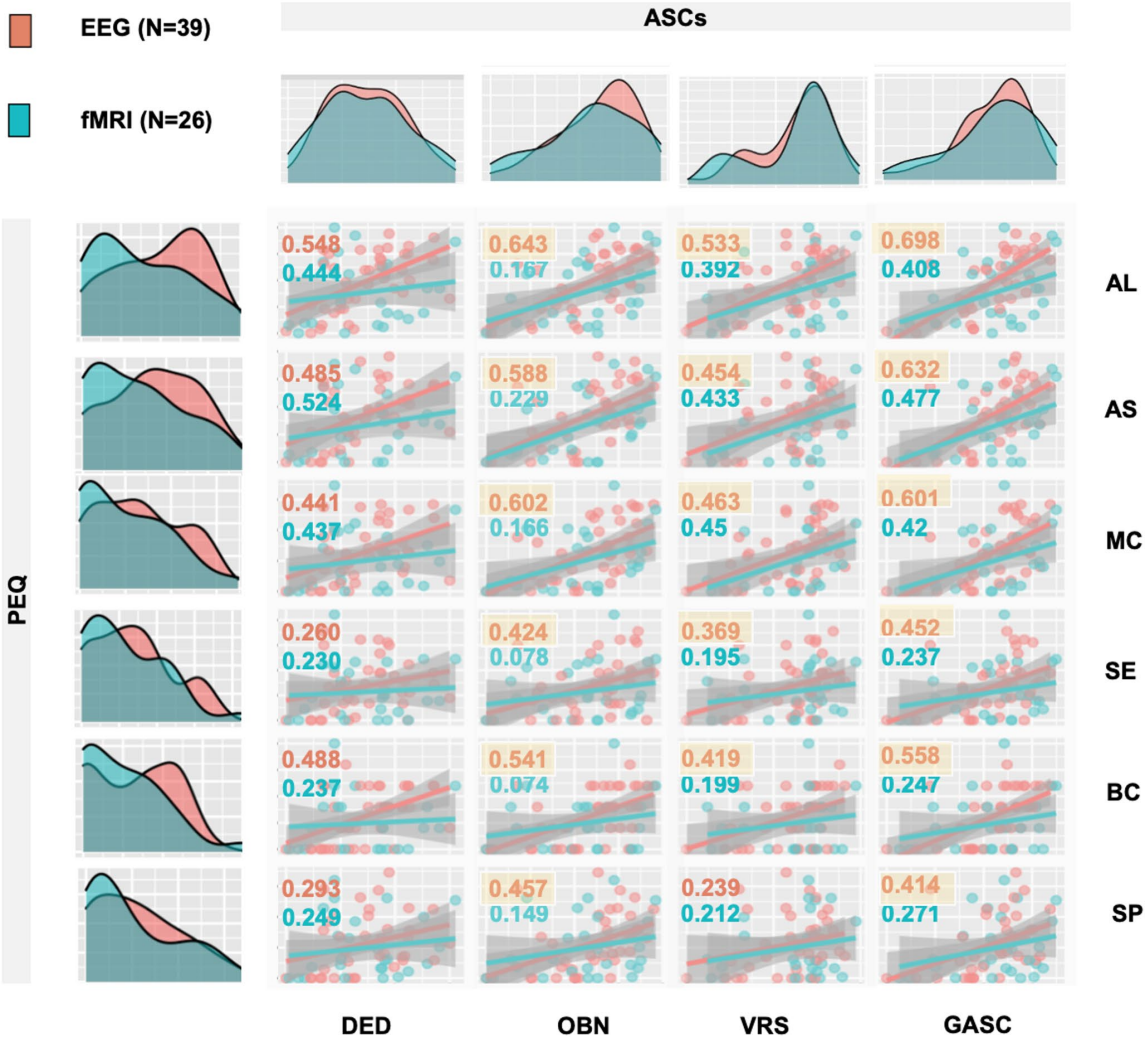
Pearson correlations between Altered States of Consciousness Scales (ASCs) domains (columns) and Persisting Effects Questionnaire (PEQ subscales) (rows) are shown for the EEG arm (*N* = 39, red) and fMRI arm (*N* = 26, blue). R-values are displayed numerically in the upper left corner of each cell (red for EEG, blue for fMRI). Statistically significant correlations after Benjamini–Hochberg correction are highlighted in yellow. Notably, significant correlations were predominantly observed in the EEG arm, particularly for Oceanic Boundlessness (OBN), General Altered States of Consciousness (GASC), and Visionary Restructuralization (VRS) domains. Abbreviations: AL - Attitudes towards life, AS - Attitudes toward self, ASCs - Altered States of Consciousness Scales, BC - Behavioural changes, DED - Dread of Ego Dissolution, EEG - Electroencephalography, fMRI - Functional Magnetic Resonance Imaging, GASC - General Altered States of Consciousness, MC - Mood changes, OBN - Oceanic Boundlessness, PEQ - Persisting Effects Questionnaire, SEM - Standard Error of the Mean, SE - Social effects, SP - Spirituality, VAS - Visual Analogue Scale, VRS - Visionary Restructuralization. Data were collected at the National Institute of Mental Health (formerly the Prague Psychiatric Center) between 2013 and 2020. Clinical trial registered under EudraCT No. 2012-004579-37

Correlation matrix for EEG and fMRI shows that in the EEG arm, all ASCs subscales were positively correlated with long-term positive PEQ outcomes (Fig. 3). Except for DED, all correlations survived corrections. In the fMRI arm, the effects were less pronounced and none survived corrections.

### Visual analogue scale (VAS)

**Fig. 4:**
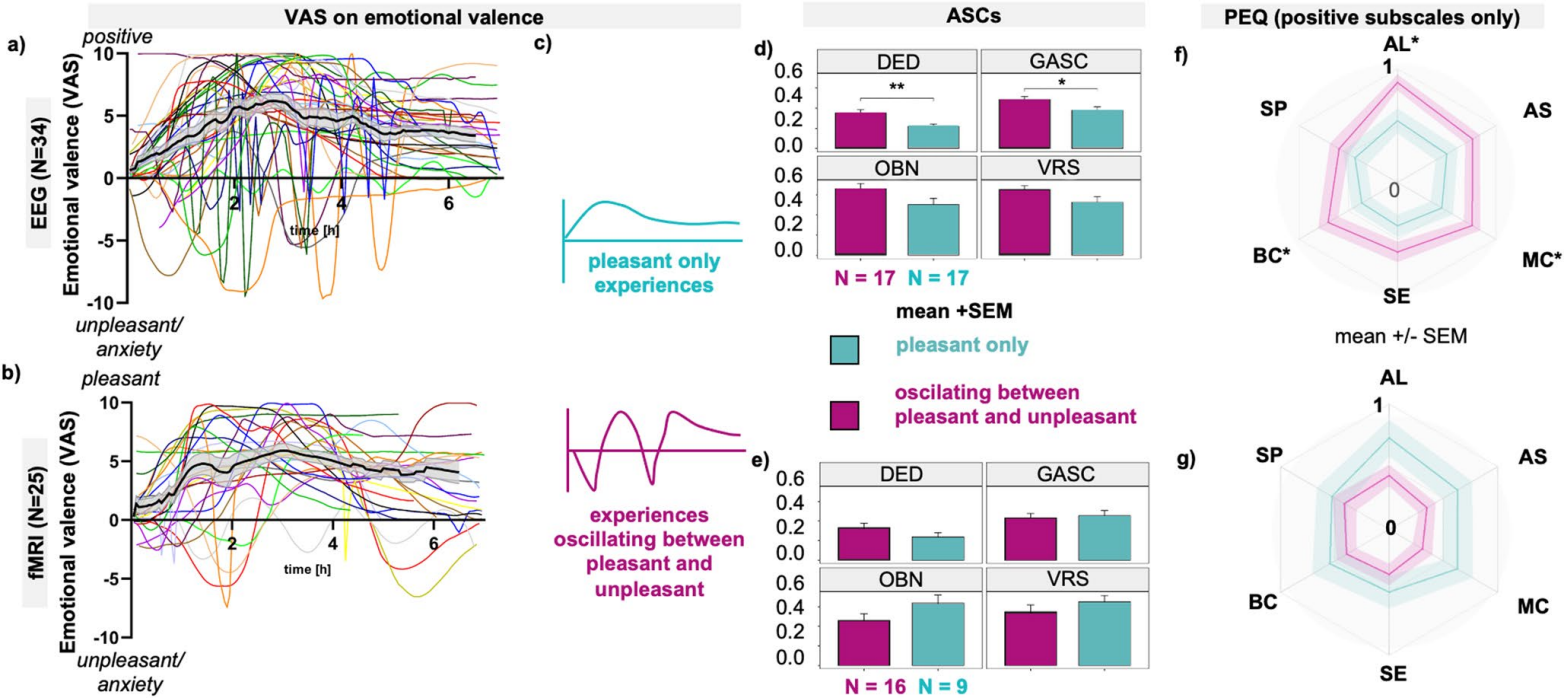
Participants experienced either only pleasant experiences or oscillated between pleasant and unpleasant, with resolution towards pleasant by 6th hour. One participant experienced only unpleasant emotion (EEG session). **(a)** EEG arm (*N* = 34) and **(b)** fMRI arm (*N* = 25) data show time-resolved emotional valence ratings during the acute psychedelic experience, assessed using the Visual Analogue Scale (VAS; range − 10 to + 10). Each line represents one participant. The black line shows the mean, the grey border shows ± SEM. **(c)** Participants were divided into two groups based on VAS patterns: “pleasant only” and “oscillating between pleasant and unpleasant. **d**,** e)** Comparison of Altered States of Consciousness Scales (ASCs) subscales between the two groups shows significant differences in Dread of Ego Dissolution (DED) and General Altered States of Consciousness (GASC) in **(d)** EEG arm, but not in **(e)** fMRI arm. **f**,** g)** Comparison of Persisting Effects Questionnaire changes (PEQ) subscales between the two groups shows significant differences in positive Attitudes about life, Mood changes, and Behavioural changes in **(f)** EEG arm, but not in **(g)** fMRI arm. Statistical comparison was performed using an independent-sample Student’s t-test. * indicates *p* < 0.05; ** indicates *p* < 0.005 (uncorrected). Abbreviations: AL - Attitudes towards life, AS - Attitudes toward self, ASCs - Altered States of Consciousness Scales, BC - Behavioural changes, DED - Dread of Ego Dissolution, EEG - Electroencephalography, fMRI - Functional Magnetic Resonance Imaging, GASC - General Altered States of Consciousness, MC - Mood changes, OBN - Oceanic Boundlessness, PEQ - Persisting Effects Questionnaire, SE - Social effects, SP - Spirituality, VAS - Visual Analogue Scale, VRS - Visionary Restructuralization. Data were collected at the National Institute of Mental Health (formerly the Prague Psychiatric Center) between 2013 and 2020. Clinical trial registered under EudraCT No. 2012-004579-37

Assessment of the individual emotional states showed that there were three types of experience: (1) pleasant only (EEG: *n* = 17; fMRI: *n* = 9), (2) oscillating between pleasant and unpleasant (EEG: *n* = 17; fMRI: *n* = 16) and (3) unpleasant only (EEG: *n* = 1). At the end of the sessions, mean experiences converged towards a pleasant emotional stateat the individual level, the majority ended in a pleasant or returned to baseline, and three subjects in the fMRI arm ended in a slightly unpleasant emotional state (Fig. 4a, b).

### Additional exploratory analysis

The RIPE was calculated from the VAS scores. For example, a score of 1 can be approximated as 100% of the experience being positive, whereas 0.83 can be approximated as 83% of the experience being positive. RIPE allowed us to divide the sample into RIPE equivalents above and below a given threshold. We chose an arbitrary threshold of 0.98, which conveniently divides the sample into two groups of the same size. Descriptive statistics for the relative index of positive experience (RIPE) are illustrated in Table S5 in the online supplement.

By dividing the sample in this way (Fig. 4c), the ASCs and PEQ subscales were analysed. In the EEG arm, participants who experienced both pleasant and unpleasant emotions had significantly higher DED and GASC, but significantly higher long-term effects (attitudes about self, positive behavioural changes, and positive changes in mood, Fig. 4d, f). We did not see this effect in the fMRI branch (Fig. 4e, g). *P-values* were not corrected.

Correlations between peak/end scores showed mild positive correlations (uncorrected) for of subscales on the PEQ (Fig. S5 in the online supplement).

### Responders and non-responders

There were 5 non-responders to psilocybin. Non-response to psilocybin was defined as the ASCs’ scores close to the mean of the placebo group or scores below the 1st quartile – 1.5 inter-quartile ratio (IQR). One non-responder had very low serum levels of psilocybin compared to a comparable subject (a women around 50 years old, with the same dose of 18 mg of psilocybin).

There we 4 placebo responders, with response defined as scores close to the mean of the psilocybin group, or above the 3rd quartile + 1.5 IQR. The placebo responders show two types of experience. First, a change in a state of consciousness for up to an hour and a half, followed by a return to normality. This may be explained by the effect of expectancy and the environment, followed by a functional unblinding, and this experience is seen only in the first session of each arm, where participants were uncertain about condition assignment. In the second type of experience, which may be called the true placebo response, subjects experienced psychedelic effects throughout the session (Table S4 in the online supplement).

At the time of the study design, the nocebo effect was not yet an understood concept, and from available data, we cannot infer the occurrence of a nocebo effect. This may be because, in any case, participants knew they would receive psilocybin in one of the conditions.

## Discussion

This study has demonstrated that the overall intensity of the psilocybin experience, at a dose of 0.26 mg/kg, is of moderate level, encompassing the entire range of effects/dimensions as characterized by the ASCs, with notably lower levels of DED compared to OBN and VRS subscales. Utilizing VAS we observed participants with exclusively positive effects, those with fluctuating experiences including challenging or anxious moments, and only one individual who had a solely difficult experience on one occasion. Predominantly, participants ended their sessions in either a neutral or positive emotional state, with only three reporting a marginally negative mood at the end. The experiences were predominantly rated as having lasting positive or neutral impacts on well-being, with minimal negative effects that did not reach clinical or statistical significance. Our findings indicate no significant influence of sex, previous psilocybin exposure, or setting (EEG vs. fMRI) on the ASCs and PEQ outcomes. Intriguingly, all ASCs dimensions, including DED, correlated positively with persistent positive effects, suggesting that the overall intensity of the experience is a key driver of this relationship. Exploratory analyses further imply that the nature of the peak experience, in conjunction with the emotional state at the session’s end, may serve as an indicator for predicting the long-term impact of individual psychedelic experiences.

### ASCs, VAS, PEQ

The overall effects on ASCs and PEQ are in line with previously published data on comparable items [5, 6, 21, 38, 45, 58] and correspond to lower medium/optimal doses of psilocybin [59–61], with about half of the subjects having a full psychedelic experience. The added value of our study is that we found no difference in the ASCs and PEQ between naive and experienced participants, nor between the first and second psilocybin administration. Furthermore, there were no differences in how many of the subjects in our sample rated the experience as one of the most significant in their lives, regardless of their previous experiences. This is partly in contrast to Studerus et al. 2012 [44], where psychedelic naive subjects reported stronger effects on Disembodiment, VRS, and Changed Meaning of Percepts of the 5D-ASC. One explanation for our observations could be that all participants may have perceived the experience as equally intense due to the overall novelty of the setting, which would mask their previous experience. Also, most of the experienced subjects had used psilocybin in a recreational setting, where lower doses might be expected [62]. Our results are also partly in contrast to Haijen et al. 2018 [45], who described that less experienced users showed greater improvements in well-being. Nevertheless, this is a very important finding in the context of the potential therapeutic use of psilocybin, as it shows that the effects of psilocybin in a controlled setting do not diminish with repeated administration, providing a rationale for repeated use in a therapeutic context.

We found no significant effect of treatment order (psilocybin-first vs. placebo-first) on acute or long-term outcomes. This suggests that the order in which treatments were administered did not influence the subjective experience or its persisting effects.

The fact that we did not find an effect of sex on acute effects is consistent with previous work by [44]. However, it is important to note that women in our study generally used lower doses of psilocybin than men (approximately 3 mg less) based on their weight, yet still achieved comparable intensities of psychedelic experience. This is interesting in the context of the work of Garcia-Romeu et al. 2021 [60], who showed no significant effect of dose-adjusted effects, including weight and sex variables. Also, women in our protocol were not dosed during the menstrual cycle, so we partially eliminated response fluctuations because of the different phases of the cycle, which has been shown in animals to interact with psilocin effects [51].

As mentioned in the Methods section, we decided at the time of designing the study protocol that the EEG arm would precede the fMRI arm for safety reasons. In support of this, Studerus [44] has also described that being placed in a Positron Emission Tomography scanner (PET) while intoxicated is associated with negative subjective experiences. Not all subjects indeed decided to proceed to the fMRI, at least some of them changed their minds and reported that they were afraid of being locked in the MRI tunnel during the experience. On the other hand, our data showed that among those who underwent all sessions, there was no significant difference in ASCs between the two settings, including the DED scale. It is worth noting that the neuroimaging nature of the study and the series of neurocognitive tasks may have prevented subjects from fully enjoying the experience. Thus, the phenomenology in our study may differ from what people might experience in a naturalistic or therapeutic setting, where the experience is usually uninterrupted by such disturbances [63].

Interestingly, Studerus and Haijen [44, 45] showed that young age was also associated with negative subjective experiences. We did not observe such an effect, partly because the subjects were carefully selected as healthy volunteers and the minimum age allowed by the protocol was 28 years, with a mean age of 35.8 years.

A detailed examination of the emotional valence of the experience using the VAS showed, in contrast to the ASCs, a variance in the dynamics of the effects, with some subjects having experiences that oscillated between pleasant and unpleasant emotional states and others having only pleasant experiences. It is noteworthy that out of almost 70 experiences, only one was unpleasant throughout the session and could be described as an overall difficult experience. On the other hand, many of the subjects had only pleasant experiences in both conditions, and at the same time, almost all of them ended up in a pleasant mood state at the end of the session. We interpret this finding that all sessions were well guided, even in a relatively difficult neuroimaging setting [44] with many demanding and disturbing tasks, such as time in fMRI. At the same time, it shows that the ASCs is a very general scale that is not designed to capture the dynamics of the experience.

Positive, persisting changes in our study were consistent but less pronounced than in previously reported studies [5, 6]. This could be due to several reasons, such as the slightly lower dose in our study, the effect of the more demanding setting due to the EEG and fMRI experiments, the blood sampled from an *iv* cannula, and the neuropsychological tests in our study. It is notable that despite these differences, many of our participants (33%), as in Griffith’s study, rated the psilocybin experience as one of the top five most meaningful experiences of their lives, as the single most spiritually significant experience of their lives (15%), and as one of the top five most spiritually significant experiences (21%).

### Relationship of subjective effects of the experience (ASCs and VAS) with persisting effects (PEQ)

Our results confirmed that OBN, VRS, and GASC are significantly correlated with almost all positive subscales of PEQ, which is in line with other studies [21, 38, 58]. However, we also found positive correlations of the DED with the PEQ, which contradicts previous findings that a negative/anxious part of the experience is associated with a lack of positive/antidepressant effects [21]. However, it is important to recognize that the ASCs questionnaire is a retrospective assessment of the whole experience, neglecting the dynamics and characteristic fluctuations of the state, where DED-like experiences can switch to OBN and vice versa, and such oscillations of emotional state can occur several times during a session. As mentioned above, based on our VAS, we saw that some subjects had an overall positive experience, while others oscillated between positive and negative emotional states. In our EEG arm, it appeared that subjects with both positive and negative experiences had overall more intense experiences based on ASCs and also more positive long-term outcomes. However, as this effect was not confirmed in the fMRI arm, putting these two factors together again makes it more likely that the intensity of the experience is the main factor at play, in line with other findings [30, 32, 35, 36]. We hypothesized that aside from intensity, the end state of the experience could also influence the long-term effects. Our study found that the combination of peak experience and end-state positivity weakly correlated with lasting positive effects. Further exploration is needed to determine if individuals who undergo difficult breakthroughs during the session have a peak and end up in a positive emotional state, can continue in that positive afterglow, and vice versa. This could potentially shed light on why psychedelic experiences sometimes lead to persistent anxiety or negative effects in non-supportive environments. This underscores the importance of a safe and supportive setting [64–66].

Interestingly, significant correlations between acute subjective experiences (ASCs) and long-term effects (PEQ) were observed primarily in the EEG arm, while these associations were less pronounced in the fMRI arm. This could suggest a potential carry-over effect, as all participants underwent the EEG arm before the fMRI arm. Although we found no significant effect of treatment order (psilocybin-first vs. placebo-first) on ASCs or PEQ scores, the fixed sequence of EEG preceding fMRI does not allow us to entirely rule out the influence of arm order. Additionally, the fMRI environment is inherently more stressful due to its noise, spatial restriction, and physical demands, which may have influenced the integration of acute experience into long-term outcomes. Importantly, however, we did not observe significant differences in ASC or PEQ scores between the EEG and fMRI arms; the difference appeared only in the strength of their association.

#### Psilocybin non-responders, placebo responders, Nocebo effect

To our knowledge, no study to date has described details of non-responders and placebo responders to the acute effects of psilocybin. A closer look at non- or mild responders and placebo responders in our sample revealed differences in serum levels of psilocin in some, but not all, which could explain the variability. While the placebo effect has been described quite frequently, the non-response to psychedelic doses of psilocybin came as quite a surprise to us.

Regarding the serum levels of psilocin, it is worth noting that all subjects were asked to remain food free since dinner, but we did not control for what they ate, and some foods could theoretically interact for longer. Furthermore, we do not know much about other factors such as the microbiome, fast and slow metabolisers, etc., which could also contribute. Interestingly, one of the low responders who felt nothing in the two sessions, despite having adequate psilocybin serum levels, points to an individual who most likely has a lower sensitivity to psilocybin.

In our sample of 40 initially enrolled subjects, 10% appeared to be placebo responders (Table S4 in the online supplement). Interestingly, two subjects in the EEG group reported a placebo effect on ASCs at the second session. This is difficult to explain, as the unblinding effect after the first active session was typical for most subjects. It could theoretically reflect the fact that the subjects were listening to music or sounds during the EEG arm, which could lead to scoring on the auditory questions resulting in an increase in the VRS of ASCs, or the fact that the subject felt relaxed and in a positive mood throughout the session, which could feed the OBN. Interestingly, two participants who responded to the placebo reported long-term positive outcomes that appeared to be driven by the intensity of their acute experience, as measured by the ASCs. Although we cannot statistically substantiate this observation, one can speculate that the psychedelic experience itself, regardless of the mechanism by which it was induced, could be driving long-term positive effects.

Finally, we did not observe any nocebo effect. Our sample consisted of healthy volunteers, and given the fact that psilocybin generally causes minimal serious adverse effects (apart from the difficult psychedelic experience, which is debatable as to whether it is an adverse effect), it would be unlikely to detect such an effect.

### Limitations

A common limitation of psychedelic trials is the process of blinding and unblinding [67]. At the time the study was designed, it adhered to the standards of placebo-controlled trials, something that would be addressed today with an active placebo, or different doses of drug, or collection of unblinding data in a questionnaire. However, given our results showing that naivety and repeated dosing did not affect acute or long-term effects, our results are minimally biased and are in agreement with the results of Goodwin et al. 2022 [1] where naïve participants were unable to subjectively discriminate between doses, but a dose-dependent response was still observed.

An obvious limitation of our study is the lower statistical power within the fMRI arm, where we did not expect so many people to withdraw from this part of the study. However, it should be noted that the results presented here are mostly exploratory, and the study was not powered for them.

Finally, an important factor arising from non-responders and placebo/nocebo responders is that data in small sample studies can be affected by these subjects. Placebo responders had low scores for both acute and persistent effects. While this was not a major problem when comparing the whole sample, it could be a problem when we split the sample in the VAS analysis. Our sample was too small to study these two groups separately, which would probably be the best solution. Therefore, in future larger trials, non-responders and placebo/nocebo responders should also be given more attention and studied independently, as this may open new ways of identifying them before the start of the trial/treatment.

## Conclusions and implications

This study reinforces existing literature by demonstrating that a healthy, non-clinical cohort can derive substantial benefits from psilocybin experiences. These benefits include enduring positive shifts in attitudes towards self and the environment, enhanced personal meaning, spiritual significance, and overall well-being. Notably, these effects persisted following a second exposure, despite an inter-dosing interval of approximately 1.5 years. Moments of anxiety and challenge, as indicated by the DED scale and the VAS, appear to be integral components of the psychedelic experience. Such challenging experiences do not invariably lead to negative long-term outcomes; in fact, they may contribute positively in a controlled setting. This suggests that anxiety, as a facet of the psychedelic process, should not be excessively mitigated. The depth and significance of the experience are influenced by the set and setting, which could explain the relatively lower PEQ scores in our neuroimaging study compared to others. Importantly, our findings carry clinical implications, bolstering the potential of repeated psilocybin administrations in psychedelic-assisted therapy. This benefit seems to extend to patients regardless of their previous experience with psychedelics or with biological sex.

## Electronic supplementary material

Below is the link to the electronic supplementary material.

Supplementary Material 1

## Data Availability

The datasets generated during and/or analysed during the current study are available from the corresponding author upon reasonable request.

## Abbreviations

5D-ASC: Five Dimensional Altered States of Consciousness
APZ: Aussergewöhnliche Psychische Zustände (original German name for ASCs)
ASCs: Altered States of Consciousness Scales
AUC: Area under the curve
pAUC: Positive area under the curve
BH: Benjamini-Hochberg correction
DED: Dread of Ego Dissolution
DV: Dependent variable
EEG: Electroencephalography
F: Female
fMRI: Functional Magnetic Resonance Imaging
GASC: General Altered States of Consciousness
GC-MS: Gas chromatography–mass spectrometry
IKEM: Institute of Clinical and Experimental Medicine in Prague, Czechia
IQR: Inter-quartile ratio
LC-MS: Liquid chromatography–mass spectrometry
M: Male
MEQ: Mystical Experience Questionnaire
MINI v 5.0: Mini-International Neuropsychiatric Interview version 5.0
MMPI-2: Minnesota Multiphasic Personality Inventory
NMDA: N-methyl-D-aspartic acid
OBN: Oceanic Boundlessness
PEQ: Persisting Effects Questionnaire
PET: Positron Emission Tomography
RIPE: Relative Index of Positive Experience
SEM: Standard Error of the Mean
SD: Standard deviation
VAS: Visual Analogue Scale
VRS: Visionary Restructuralization

## Footnotes

1 Order in which psilocybin and placebo were administered.
