# Supplementary material for: The phenomenology of psilocybin’s experience mediates subsequent persistent psychological effects independently of sex, previous experience, or setting
Source: Pharmacol Rep. 2025 Jun 16;77(4):1024–39. doi: 10.1007/s43440-025-00742-5 (PMC12241220; doi:10.1007/s43440-025-00742-5)
Supplement: Supplementary file 1 — Supplementary Material 1 [file 43440_2025_742_MOESM1_ESM.docx]

# **Online Supplement**

**Table S1**

| Inclusion Criteria |
| --- |
| a) Men and women ages 28 to 65 |
| b) Healthy volunteers with no psychiatric history (mental illnesses included in ICD 10 F0.X - F99.X) |
| c) No family history of psychotic disorders up to second-degree relatives |
| Exclusion Criteria: |
| a) Pregnancy |
| b) Intracranial hypertension, arterial hypertension or pulmonary hypertension |
| c) Stroke |
| d) Congestive heart failure |
| e) Pacemaker |
| f) Metal clamp in the head and/or face |
| g) Celiac disease |
| h) Left-handedness |
| i) Usage of any medication on daily basis except for contraception |
| j) Person in dependent position – students of medical faculties |
| k) Person in dependent position – students of other faculties younger than 28 |
| Criteria for premature termination of participation in this study: |
| a) Participant can cancel his participation anytime without giving any reason |
| b) Changes in health state including use of pharmacological substances |
| c) Changes in psychic state including traumatic events last 3 months (eg. death in family etc.) |
| d) Pregnancy |
| e) Significant side effects that are related to assessments in this study |

**Table S1** Inclusion and exclusion criteria applied prior to study enrolment.

Study was conducted at the National Institute of Mental Health (formerly the Prague Psychiatric Center) between 2013 and 2020. Clinical trial registered under EudraCT No. 2012-004579-37.

**Table S2 Altered states of consciousness scale (ASCs): Mean (SD)**

|  |  | DED | OBN | VRS | GASC |
| --- | --- | --- | --- | --- | --- |
| EEG  (N=39) | psilocybin | 28.7 (14.1) | 58.9 (23.8) | 60.1 (20.9) | 45,3 (19) |
|  | placebo | 2.89 (5.32) | 6.05 (11.6) | 3 (8.08) | 3.95 (7.38) |
|  |  |  |  |  |  |
| fMRI  (N=26) | psilocybin | 29.1 (16.3) | 55.6 (27.5) | 60.9 (23.9) | 44.8 (17.5) |
|  | placebo | 1.74 (3.27) | 2.49 (4.08) | 1.44 (2.78) | 1,89 (3) |

**Table S2** Means and standard deviations (SD) for each subscale of Altered States of Consciousness Scales (ASCs), cross-over design.

Abbreviations: ASCs – Altered States of Consciousness Scales, DED – Dread of Ego Dissolution, EEG – Electroencephalography, fMRI – Functional Magnetic Resonance Imaging, GASC – General Altered States of Consciousness, OBN – Oceanic Boundlessness, VRS – Visionary Restructuralization

Data were collected at the National Institute of Mental Health (formerly the Prague Psychiatric Center) between 2013 and 2020. Clinical trial registered under EudraCT No. 2012-004579-37.

**Table S3 Persisting Effects Questionaire (PEQ)**

|  |  | Attitudes about life | Attitudes about self | Mood changes | Behavior changes | Altruistic/positive social effects | Increased spirituality |
| --- | --- | --- | --- | --- | --- | --- | --- |
| EEG  (N=39) | psilocybin | 39.1 (22.2) | 34.5 (21.9) | 32.3 (23.6) | 27.2 (21.2) | 31.3 (25.5) | 28.2 (24.1) |
|  | placebo | 10.7 (14.9) | 9.6 (15.9) | 7.52 (11.9) | 7.24 (13.3) | 6.15 (17.3) | 8.79 (19) |
|  |  |  |  |  |  |  |  |
| fMRI  (N=26) | psilocybin | 30.9 (24.4) | 26.4 (22.8) | 25.3 (24.3) | 22.7 (21.6) | 24.6 (26.1) | 24.8 (25) |
|  | placebo | 8.52 (19.1) | 7.92 (16.8) | 8.02 (18.6) | 7.38 (16.3) | 9.29 (22.1) | 8.25 (18.6) |

**Table S3** Means and standard deviations for each subscale of Persisting Effect Questionnaire (PEQ) in EEG arm (N=39) and fMRI arm (N=26).

Abbreviations: EEG – Electroencephalography, fMRI – Functional Magnetic Resonance Imaging, PEQ – Persisting Effects Questionnaire

Data were collected at the National Institute of Mental Health (formerly the Prague Psychiatric Center) between 2013 and 2020. Clinical trial registered under EudraCT No. 2012-004579-37.

**Table S4 Placebo responders and psilocybin non-responders, detailed characteristics of individuals**

|  | Sex (M/F) | age | weigh (kg) | dose (mg) | experienced / naïve | mental healthcare / other | arm | session | GASC | PEQ Positive | psilocin plasma levels (ng/ml) at 120 min | session | GASC | PEQ Positive |
| --- | --- | --- | --- | --- | --- | --- | --- | --- | --- | --- | --- | --- | --- | --- |
|  |  |  |  |  |  |  |  |  |  |  |  |  |  |  |
| placebo responders | M | 30 | 75 | 19 | E | O | EEG | 1st | 33,38 | 25,82 | n.d | 2nd | 59,21 |  |
|  | F | 37 | 56 | 16 | N | O | EEG | 1st | 13,24 | 10,1 | n.d | 2nd | 60,3 | 54,04 |
|  | F | 28 | 67 | 18 | N | O | EEG | 2nd | 32,04 | 38,99 | n.d | 1st | 53,33 | 67,09 |
|  | F | 30 | 65 | 17 | N | MH | EEG | 2nd | 7,89 | 16,65 | n.d | 1st | 51,21 | 20,47 |
|  |  |  |  |  |  |  |  |  |  |  |  |  |  |  |
| psilocybin non-responders or low-responders | F | 47 | 67 | 17 | N | O | EEG | 1st | 0 | 0,26 | 23 | 2nd | 0 | 0 |
|  | F | 28 | 60 | 16 | E | MH | EEG | 1st | 17,96 | 57,34 | 12 | 2nd | 0,46 | 56,76 |
|  | M | 28 | 72 | 19 | E | O | EEG | 2nd | 12,18 | 0 | *NA* | 1st | 2,78 | 0 |
|  | M* | 37 | 77 | 20 | E | MH | EEG | 2nd | 20,72 | 1,03 | 25 | 1st | 0,85 | 1,63 |
|  | M | 33 | 56 | 16 | E | O | fMRI | 1st | 19,15 | 21,18 | *NA* | 2nd | 11,75 | 0,26 |
|  | M* | 38 | 79 | 20 | E | MH | fMRI | 1st | 20,1 | 11,05 | 12 | 2nd | 0 | 0,51 |
|  | ** identical subjects* | |  |  |  |  |  |  |  |  |  |  |  |  |

**Table S4** Characteristics of placebo responders, and psilocybin non-responders/low responders based on Altered States of Consciousness Scales (ASCs). Placebo respondent's scores equal to the psilocybin group average or above third quartile + 1.5 * interquartile-ratio (IQR), and non-responders/low-responders scores equal to placebo average or below first quartie - 1.5 * IQR. PEQ positive average is an average of positive items on the PEQ scale. For comparison, in grey cross-over scores added.

Abbreviations: ASCs - Altered States of Consciousness Scales, E – experienced, with previous experience with psychedelics, EEG - Electroencephalography, F – Female, fMRI - Functional Magnetic Resonance Imaging, GASC - General Altered States of Consciousness, M – Male, MH – Mental health care, N – Naïve, without previous experience with psychedelics, NA - Not available, n.d. - Not detected, O – Other occupation, PEQ - Persisting Effects Questionnaire

Data were collected at the National Institute of Mental Health (formerly the Prague Psychiatric Center) between 2013 and 2020. Clinical trial registered under EudraCT No. 2012-004579-37

**Table S5 Descriptive statistics for** **the relative index of positive experience (RIPE)**

|  | Valence | Mean | SD | Median | Min | Max | Range |
| --- | --- | --- | --- | --- | --- | --- | --- |
| EEG  (N=34) | Pleasant only  N= 17 | 1 | 0 | 1 | 0.99 | 1 | 0.01 |
|  | Oscillating  N=17 | 0.81 | 0.27 | 0.93 | 0 | 0.99 | 0.99 |
| fMRI  (N=25) | Pleasant only  N=9 | 1 | 0 | 1 | 0.99 | 1 | 0.01 |
|  | Oscillating  N=16 | 0.89 | 0.13 | 0.95 | 0.53 | 0.98 | 0.45 |

**Table S5** Descriptive statistics of the Relative Index of Positive Experience (RIPE) after psilocybin administration in participants reporting either only pleasant or oscillating between pleasant and unpleasant emotional valence. RIPE values range from 0 to 1, with higher values indicating more consistently positive emotional responses. Data are presented separately for the EEG (N = 17) and fMRI (N = 13) study arms. Presented values include mean, standard deviation (SD), median, minimum, maximum, and range.

Abbreviations: EEG - Electroencephalography, fMRI - Functional Magnetic Resonance Imaging, RIPE - Relative index of positive experience, SD - Standard deviation

Data were collected at the National Institute of Mental Health (formerly the Prague Psychiatric Center) between 2013 and 2020. Clinical trial registered under EudraCT No. 2012-004579-37.

**Figure S5 Visualisation of VAS scale on emotional valence with psilocin levels and Peak + end point and correlations**


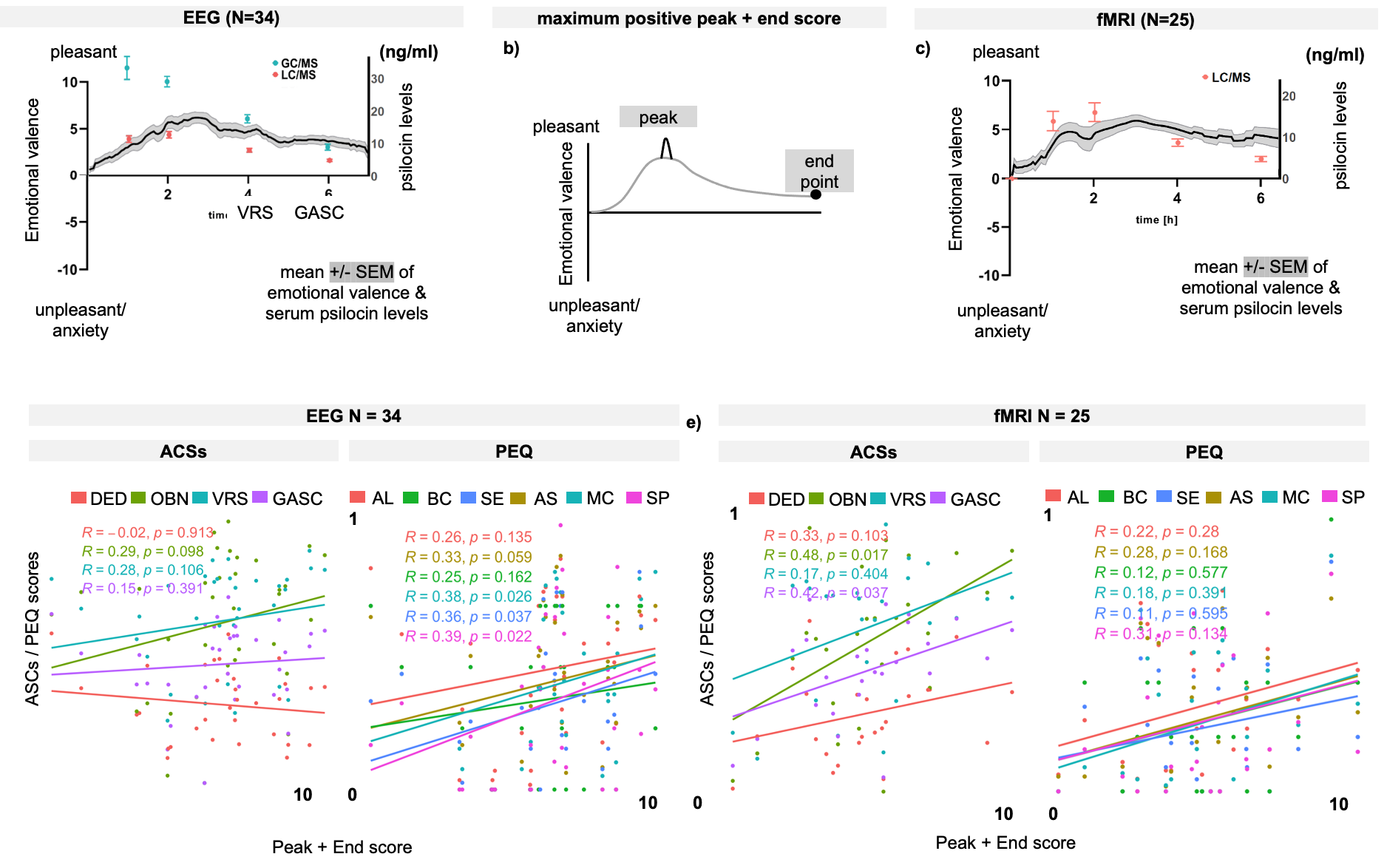


**Fig. S5 a, c)** Visualization of Visual Analogue Scale (VAS) on emotional valence with psilocin levels measured by (GC/MS and/or LC/MS) **in a)** EEG and **c)** fMRI arms. **b)** Schematic visualization of peak + end point hypothesis: individual peak valence and the last recorded valence were summed to capture overall positive affect during the session. **d,e)** Pearson correlation coefficients of Altered States of Consciousness Scales (ASCs) and Persisting Effects Questionnaire (PEQ) subscales with the „maximum positive peak + last score“ within d) EEG arm and e) fMRI arm. *P-values* uncorrected.

**Abbreviations:** AL - Attitudes towards life, AS - Attitudes toward self, ASCs - Altered States of Consciousness Scales, BC - Behavioural changes, DED - Dread of Ego Dissolution, EEG - Electroencephalography, fMRI - Functional Magnetic Resonance Imaging, GASC - General Altered States of Consciousness, GC-MS - Gas chromatography–mass spectrometry, LC-MS - Liquid chromatography–mass spectrometry, MC = Mood changes, OBN - Oceanic Boundlessness, PEQ - Persisting Effects Questionnaire, SEM - Standard Error of the Mean, SE - Social effects, SEM - Standart Error of the Mean, SP - Spirituality, VAS - Visual Analogue Scale, VRS - Visionary Restructuralization

Data were collected at the National Institute of Mental Health (formerly the Prague Psychiatric Center) between 2013 and 2020. Clinical trial registered under EudraCT No. 2012-004579-37.
